# Serological diagnosis of autoimmune bullous skin diseases: Prospective comparison of the BIOCHIP mosaic-based indirect immunofluorescence technique with the conventional multi-step single test strategy

**DOI:** 10.1186/1750-1172-7-49

**Published:** 2012-08-09

**Authors:** Nina van Beek, Kristin Rentzsch, Christian Probst, Lars Komorowski, Michael Kasperkiewicz, Kai Fechner, Inga M Bloecker, Detlef Zillikens, Winfried Stöcker, Enno Schmidt

**Affiliations:** 1 Department of Dermatology, University of Luebeck, Luebeck, Germany; 2 Institute of Experimental Immunology, EUROIMMUN AG, Luebeck, Germany; 3 Comprehensive Center for Inflammation Medicine (CCIM), University of Luebeck, Luebeck, Germany

## Abstract

**Background:**

Various antigen-specific immunoassays are available for the serological diagnosis of autoimmune bullous diseases. However, a spectrum of different tissue-based and monovalent antigen-specific assays is required to establish the diagnosis. BIOCHIP mosaics consisting of different antigen substrates allow polyvalent immunofluorescence (IF) tests and provide antibody profiles in a single incubation.

**Methods:**

Slides for indirect IF were prepared, containing BIOCHIPS with the following test substrates in each reaction field: monkey esophagus, primate salt-split skin, antigen dots of tetrameric BP180-NC16A as well as desmoglein 1-, desmoglein 3-, and BP230gC-expressing human HEK293 cells. This BIOCHIP mosaic was probed using a large panel of sera from patients with pemphigus vulgaris (PV, n = 65), pemphigus foliaceus (PF, n = 50), bullous pemphigoid (BP, n = 42), and non-inflammatory skin diseases (n = 97) as well as from healthy blood donors (n = 100). Furthermore, to evaluate the usability in routine diagnostics, 454 consecutive sera from patients with suspected immunobullous disorders were prospectively analyzed in parallel using a) the IF BIOCHIP mosaic and b) a panel of single antibody assays as commonly used by specialized centers.

**Results:**

Using the BIOCHIP mosaic, sensitivities of the desmoglein 1-, desmoglein 3-, and NC16A-specific substrates were 90%, 98.5% and 100%, respectively. BP230 was recognized by 54% of the BP sera. Specificities ranged from 98.2% to 100% for all substrates. In the prospective study, a high agreement was found between the results obtained by the BIOCHIP mosaic and the single test panel for the diagnosis of BP, PV, PF, and sera without serum autoantibodies (Cohen’s κ between 0.88 and 0.97).

**Conclusions:**

The BIOCHIP mosaic contains sensitive and specific substrates for the indirect IF diagnosis of BP, PF, and PV. Its diagnostic accuracy is comparable with the conventional multi-step approach. The highly standardized and practical BIOCHIP mosaic will facilitate the serological diagnosis of autoimmune blistering diseases.

## Background

Autoimmune bullous disorders are characterized by autoantibodies against desmosomal proteins (in pemphigus), adhesion molecules of the dermal-epidermal junction (in pemphigoid diseases), and epidermal/ tissue transglutaminase (in dermatitis herpetiformis), respectively [1-3]. The most frequent autoimmune bullous diseases are bullous pemphigoid and pemphigus, with incidences varying considerably between geographical regions [4-8]. Incidences for BP range from 13.4- 42 new patients/ million inhabitants per year [5,6,8,9]. In a population aged 80 years and above the incidence of BP has been reported to be 150–190 new patients/million/year [6,10]. In central Europe, pemphigus is less frequent with incidences ranging from 0.6 to 6.8 new patients/million/year [5,8,11], higher incidences can be found in Southeastern Europe, the Mediterranean region, Iran and the Jewish population [7,12]. In pemphigoid gestationis and mucous membrane pemphigoid incidences of 2.0 patients/million/year were reported [6,13]. Incidences of the other entities are below 1.0/million/year.

Diagnosis relies on a combination of clinical features as well as the detection of skin-/ mucous membrane-bound and circulating autoantibodies [14,15]. The diagnostic gold standard is still the visualization of skin-/ mucous membrane-bound autoantibodies by direct immunofluorescence (IF) microscopy [16]. Advances in the identification of target antigens (summarized in Table 1) and the subsequent development of an increasing number of sensitive and specific assays for the detection of circulating autoantibodies, including Western blotting of cell-derived and recombinant forms of the target antigens, immunoprecipitation, and ELISA, allow serological diagnosis in the majority of patients [15]. Several ELISA systems using recombinant fragments of BP180, BP230, desmoglein 1, desmoglein 3, envoplakin, and type VII collagen have become commercially available and are highly valuable diagnostic tools (MBL, Nagoya, Japan and EUROIMMUN AG, Luebeck, Germany) [17-23].

**Table 1:** Overview of target antigens in immunobullous diseases and diagnostic methods used in this study

**Disease**	**Target antigen**	**Routine multi-step approach**^**1**^
**Pemphigus vulgaris**	Desmoglein 3	*Desmoglein 3 ELISA*[22]
**Pemphigus foliaceus**	Desmoglein 1	*Desmoglein 1 ELISA*[22]
**Paraneoplastic pemphigus**	Desmoglein 3	*Desmoglein 3 ELISA*[22]
	Envoplakin	*Envoplakin ELISA*[21]
	Periplakin/ Desmoplakin I/II	Immunoblot with extract of cultured HaCaT cells [21]
		Indirect IF microscopy on rat and monkey bladder
**Bullous pemphigoid**	BP180	*BP180 NC16A ELISA*[20]
	BP230	*BP230 ELISA*[51]
	Soluble ectodomain of BP180 (LAD-1)	BP180 4575 (c-terminal fragment) Immunoblot [33,63]
		LAD-1 Immunoblot [33,63]
**Pemphigoid gestationis**	BP180	*BP180 NC16A ELISA*[20]
		Complement binding test
**Linear IgA dermatosis**	Soluble ectodomain of BP180 (LAD-1)	Immunoblot with conditioned medium of cultured HaCaT cells (IgA reactivity) ) [33,63]
	BP230	*BP230 ELISA*[51]
**Lichen planus pemphigoides**	BP180	*BP180 NC16A ELISA*[51]
	BP230	*BP230 ELISA*[51]
**Mucous membrane pemphigoid**	Soluble ectodomain of BP180 (LAD-1)	Immunoblot with conditioned medium of cultured HaCaT cells (IgG and IgA reactivity) [33]
	BP180	BP180 *NC16A* ELISA [20]
	BP230	BP230 ELISA [51]
	Laminin 332	Immunoblot with extracellular matrix of cultured HaCaT cells [13,35]
**laminin γ1/anti-p200 pemphigoid**	p200 protein/ Laminin γ1	Immunoblot with extract of human dermis [34]
		Immunoblot with recombinant laminin γ1 C-term [37]
**Epidermolysis bullosa acquisita**	Type VII collagen	Immunoblot with recombinant NC1-domain of type VII collagen [36]
**Dermatitis herpetiformis**	Epidermal/tissue transglutaminase, gliadin	Transglutaminase ELISA deamidated gliadin-analogous fusion peptide-specific ELISA [32]

^1^in addition, all sera were subjected to indirect IF microscopy on human salt-split skin and monkey esophagus. The exact diagnostic algorithm applied in this study is detailed in [38].

IF, immunofluorescence; LAD-1, linear IgA dermatosis antigen 1(soluble ectodomain of BP180); BP180NC16A, targeted extracellular domain of BP180.

Usually, the determination of serum autoantibodies is a multi-step procedure comprising an initial screening step by indirect IF microscopy using frozen sections of one or two tissues followed by more specific tests that aim at identifying the target antigen(s). Indirect IF microscopy on monkey esophagus (for pemphigus) and human skin, where the dermal-epidermal junction has been split by 1 M NaCl solution (for pemphigoid diseases), has been elucidated as the most sensitive screening tests [24-27]. The subsequent elaborate identification of the target antigen varies amongst different laboratories.

Here, to facilitate the serological diagnosis of immunobullous disorders, a multiplex IF BIOCHIP mosaic has been developed that combines screening and target antigen-specific substrates in a single miniature incubation field. Validation of the BIOCHIP showed high specificity and high sensitivity for pemphigus vulgaris (PV), pemphigus foliaceus (PF), and bullous pemphigoid (BP). In the second set of experiments, a large panel of consecutive sera from suspected autoimmune blistering disease patients sent to the Laboratory for Autoimmune Diagnostics of the Dermatology Department at the University of Luebeck was analysed with both the BIOCHIP mosaic and the conventional diagnostic multi-step algorithm.

## Methods

### Patients

For the validation of the novel BIOCHIP mosaic, sera from patients with BP (n = 42), PV (n = 65), and PF (n = 50) were used. Patients were characterized by (i) the typical clinical phenotype, (ii) positive direct IF result, and (iii) serum autoantibodies against BP180 NC16A, desmoglein (Dsg) 3, and Dsg1, respectively. In addition, sera from patients with linear IgA dermatosis, non-inflammatory skin diseases (NISD), including vascular leg ulcers, basal cell carcinoma, and squamous cell carcinoma (n = 97), and healthy blood donors (HBD, n = 100), were used. All sera were anonymized before testing. For the prospective comparative study, 454 consecutive sera from patients with suspected autoimmune bullous disease were analyzed independently by experienced staff members of the diagnostic laboratories of EUROIMMUN and the dermatology department, using both the BIOCHIP mosaic and the routine multi-step diagnostic algorithm. The study was approved by the local ethic committees (10–017).

In 9 patients (PV, n = 3; PF, n = 3; BP, n = 3), sera were analyzed during the course of the disease at 3–5 different time points. Disease activity for skin lesions: score of 4, >10 lesions; score of 3, 4 to 10 lesions; score of 2, 1 to 3 lesions; score of 1, clinical remission on immunosuppressive therapy; score of 0, clinical remission without immunosuppressive therapy) [28]. Scoring was done retrospectively based on photographs and patient records; serum was drawn on the same day when photographs were taken.

### BIOCHIP mosaic

The membrane bound extracellular domains of Dsg1 (amino acids 1–569;) and Dsg3 (amino acids 1–640) were cloned as described [22] with the modification of using alternate antisense primers to include the respective coding regions for the transmembrane (TM) domains: CAA TGT CTG CAC ATA GCT CTA GGC GTC GAC TTA ATG ATG ATG ATG ATG AT (Dsg1) and ATA CTC GAG TTA ATG ATG ATG ATG ATG ATG GGT CAA CAG CAG AAG GGG GGC CAA C (Dsg3). The coding region for the C-terminal globular domain of BP230 (BP230gC; amino acids 1875–2649) was amplified from an EST cDNA (DKFZp686C04183Q, acc. no. BX475892, Imagene, Berlin, Germany) in two separate fragments to delete an intron using primer pairs ATA GTC GAC GCC ATG GAC TGT ACC TTC AAA CCA GAT TTT GAG and ATA TCG TCT CTA TCT CTA AGG GTG TCA AAA CCT TCA CC and ATA TCG TCT CAA GAT AGC TAA GAA CAA GCA GTA TG and ATA CTC GAG TAA GGA AGA ATA GTA GAG GC. The two BP230gC fragments are linked together, employing the unique BspHI site. The cDNAs were digested with the appropriate restriction enzymes and then ligated with linearized pTriEx-1 (Merck Biosciences, Darmstadt, Germany).

**Figure 1:**
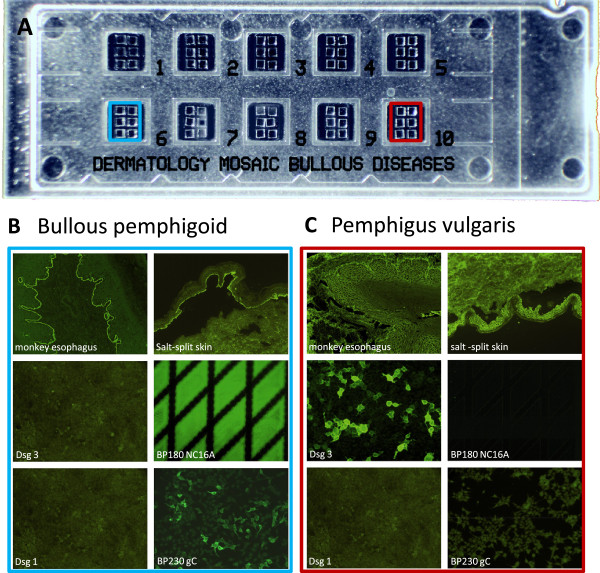
**BIOCHIP mosaic for immunobullous disorders.** (**A**) On a standard-sized slide, there are ten incubation fields each with six different BIOCHIPs. (**B**, **C**) Representative stainings after incubation with a bullous pemphigoid (**B**) and pemphigus vulgaris (**C**) serum. Desmoglein (Dsg) 1, Dsg3, and BP230gC (C-terminal globular domain of BP230) are expressed in human HEK293 cells. BP180 NC16A is directly coated on the BIOCHIP. HEK293 cells transfected with pTriEx-1 serve as negative control.

HEK293 cells (ATCC, CRL-1573) were cultivated and transfected with the pTriEx-1 constructs of Dsg1-TM, Dsg3-TM, and BP230gC as described [22] and seeded on cover slides. After cultivation for 48 hrs at 37°C, cells were fixed with formalin (Dsg) or acetone (BP230) for 10 min and stored in liquid nitrogen until further processing. Other cover glasses were coated with recombinant tetrameric NC16A (BP180-NC16A-4X), expressed as described previously [20], and with frozen tissue sections of monkey esophagus and primate 1 M NaCl-split skin. Cover glasses were automatically cut into millimeter-sized fragments (BIOCHIPs) and glued side by side on microscopy slides, resulting in multiparametric BIOCHIP mosaics (Figure 1A) for simultaneous incubation of each serum sample on all substrates.

Slides containing the BIOCHIP mosaics were incubated with serial serum dilutions (starting with 1:10 in PBS-Tween) for 30 min at room temperature, rinsed with a flush of PBS-Tween and immersed in PBS-Tween for 5 min. For detection of bound antibodies, fluorescein isothiocyanate (FITC)-conjugated goat anti-human IgA/G (EUROIMMUN) was applied for 30 minutes at room temperature, followed by washing as described before. The slides were then embedded using mounting medium (EUROIMMUN) and examined by fluorescence microscopy. Titer steps of 1:10, 1:32, 1:100, 1:320, 1:1000 and 1:3200 were obtained for correlation with disease activity in selected sera.

Diagnosis of BP was made based on reactivity with salt-split skin or monkey esophagus *and* reactivity with BP180 or BP230. In case of basal membrane zone staining without reactivity against BP180 or BP230 the diagnosis was “pemphigoid disease”. When no reactivity was seen in any of the six biochips of one incubation field, a negative result was obtained.

### Multi-step serum analysis

All samples were analyzed by experienced investigators using indirect IF microscopy on monkey esophagus and 1 M NaCl-split human skin [26] for the detection of anti-human IgG and IgA secondary antibodies (both Bio-Rad, Munich, Germany) and a MicroImaging microscope (Carl Zeiss, Jena, Germany). In case of suspected pemphigoid gestationis and paraneoplastic pemphigus, IF microscopy on salt-split human skin (complement binding [29]) and on rat/monkey bladder was performed, respectively. According to IF results and clinical suspicion, target antigen-specific assays were employed, including (i) Dsg 1-specific ELISA [22], (ii) Dsg 3-specific ELISA [22], (iii) BP180-specific ELISA [20,30], (iv) BP230-specific ELISA [19,31], (v) envoplakin-specific ELISA [21], (vi) transglutaminase-specific ELISA [32], (vii) deamidated gliadin-analogous fusion peptides-specific ELISA [32] (all EUROIMMUN; all performed following the manufacturer’s instructions), (viii) immunoblotting (IB) with the soluble ectodomain of BP180 (LAD-1) in conditioned concentrated medium of cultured HaCaT [33], (ix) IB with dermal extract [34], (x) IB with cultured HaCaT cells [21], (xi) IB with the extracellular matrix of cultured HaCaT cells [35], (xii) IB with the recombinant NC1-domian of type VII collagen [36], and (xiii) IB with the recombinant C-terminus of laminin γ1 [37] (Table 1). In all sera with suspected BP, the BP180 NC16A ELISA was used. The exact diagnostic algorithm of the autoimmune laboratory, Department of Dermatology, University of Luebeck, applied in this study is detailed in [38].

Diagnosis was based on (i) a positive ELISA, (ii) a positive indirect IF in combination with a positive IB result, or (iii) positive results in 2 different IBs. A serum was evaluated as “negative” when all tests were unreactive or showed only a weak staining at a dilution of 1:10. For post hoc analysis of sera with discordant results compared to the BIOCHIP mosaic, additional analyses were performed, including Dsg 1-, Dsg 3-, BP180-, and BP230-specific ELISA (MBL) [17,19,30].

### Statistics

To determine the sensitivity and specificity of the BIOCHIPs used in the evaluated mosaic, receiver operating curves (ROC) were analyzed. Confidence intervals, sensitivity and specificity were calculated using SigmaPlot 11.0 analysis software (SSI, San Jose, CA, USA). Cohen’s Kappa was calculated with Gnu R open access software (R Development Core Team 2009; R Foundation for Statistical Computing, Vienna, Austria, volume 2.13, package “irr”) to evaluate the inter-rate analytical agreement between the BIOCHIP mosaic and the multi-step procedure [39]. κ values of 0.41-0.60 are rated as moderate concordance, κ values of 0.61-0.80 as substantial and of 0.81-1.00 almost perfect concordance [40]. Sera were anonymized before analysis.

## Results

### Validation of the BIOCHIP mosaic

Anti-basal membrane zone reactivity was seen in 41 of 42 BP sera (98.8%). Antibodies against tetrameric BP180 NC16A and BP230gC were found in 42 (100%) and 23 (54.8%) BP sera, respectively. Specificities were calculated to be 98.8%, 98.2%, and 100% (Table 2). Staining of a representative BP serum is shown in Figure 1B.

**Table 2:** Characteristics of pemphigoid disease-related substrates used in the BIOCHIP mosaic

**Disease**	**BP180-NC16A-4X**	**BP230gC**
**Bullous pemphigoid**	**42/42**	**23/42**
Sensitivity	100%	54.8%
95%CI	91.6-100%	38.7-70.2%
Specificity	98.2%	100%
95%CI	96.7-99.3%	98.9-100%
**Pemphigus vulgaris**	**0/65**	**0/65**
**Pemphigus foliaceus**	**0/50**	**0/50**
**Linear IgA disease**	**0/18**	**0/18**
**HBD**	**1/100**	**0/100**
**NISD**	**7/97**	**0/97**

BP230gC, c-terminal globular domain of BP230; HBD, healthy blood donors; NISD, non-inflammatory skin diseases; 95%CI, 95% confidence intervals.

Intercellular epithelial staining on monkey esophagus by indirect IF was observed in 65 PV and 49 PF sera resulting in sensitivities of 100% and 98.0%, respectively. Anti-Dsg3 antibodies were detected in 64 of 65 (98.5%) PV sera, in 34 (52.3%) of these sera, additional anti-Dsg1 reactivity was found. Staining of a representative PV serum is shown in Figure 1C. Anti-Dsg1 reactivity was present in 45 of 50 (90%) PF sera. Specificities for indirect IF microscopy on monkey esophagus, anti-Dsg1 reactivity, and anti-Dsg3 reactivity were 89.1%, 100%, and 99.6%, respectively (Table 3).

**Table 3:** Characteristics of pemphigus-related substrates used in the BIOCHIP mosaic

**Disease**	**Desmoglein 1**	**Desmoglein 3**
**Pemphigus vulgaris**	**33/65**	**64/65**
Sensitivity	52.3	98.5
95%CI	39.5% to 64.9%	91.7% to 100%
Specificity	100	99.6%
95%CI	98.6% to 100%	98.6% to 100%
**Pemphigus foliaceus**	**45/50**	**3/50**
Sensitivity %	90%	not applicable
95%CI	78.2% to 96.7%	
Specificity %	100%	99.6%
95%CI	98.6% to 100%	97.9% to 100%
**Linear IgA disease**	**0/18**	**0/18**
**Bullous pemphigoid**	**0/42**	**1/42**
**HBD**	**0/100**	**0/100**
**NISD**	**0/97**	**0/97**

HBD, healthy blood donors; NISD, non-inflammatory skin diseases; 95%CI, 95% confidence intervals.

### Correlation of serum autoantibody levels with disease activity

**Figure 2:**
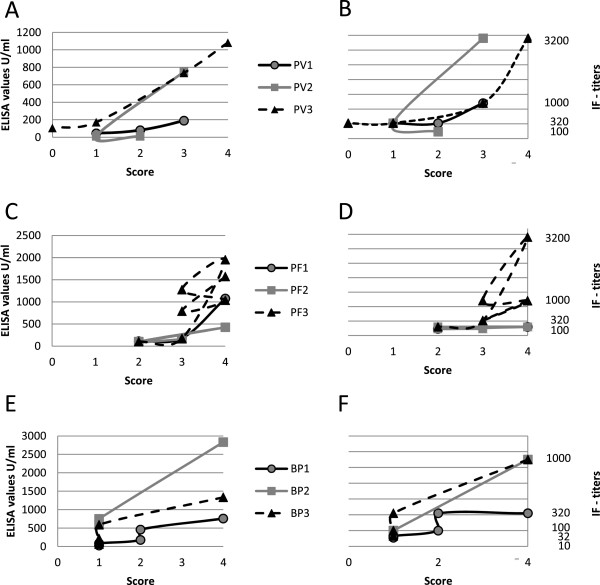
**Correlation of serum autoantibody levels with disease activity.** Values by conventional ELISA (Dsg3 (**A**), Dsg1 (**C**), BP180 (**E**)) paralleled indirect immunofluorescence titers by the BIOCHIP mosaic (Dsg3 (**B**), Dsg1 (**D**), BP180 (**E**)) obtained by testing sera from patients with pemphigus vulgaris (PV1-3) (**A**,**B**), pemphigus foliaceus (PF1-3) (**C**,**D**), and bullous pemphigoid (BP1-3) (**E**,**F**). Both ELISA values and indirect immunofluorescence titers appeared to correlate with disease activity determined by a clinical score (4, more than 10 lesions; 3, 4–10 lesions; 2, 1–3 lesions; 1, clinical remission on immunosuppressive therapy; 0 clinical remission without immunosuppressive therapy). Dsg, desmoglein; IF, immunofluorescence.

Sera of nine patients (BP, n = 3; PF, n = 3; PV, n = 3) taken during the course of the disease were analyzed by both the BIOCHIP mosaic and conventional ELISA. IF titers by the novel biochip paralleled both ELISA values and disease activity (Figure 2).

### Prospective study on the diagnostic agreement between the BIOCHIP mosaic and the conventional multi-step procedure

**Figure 3:**
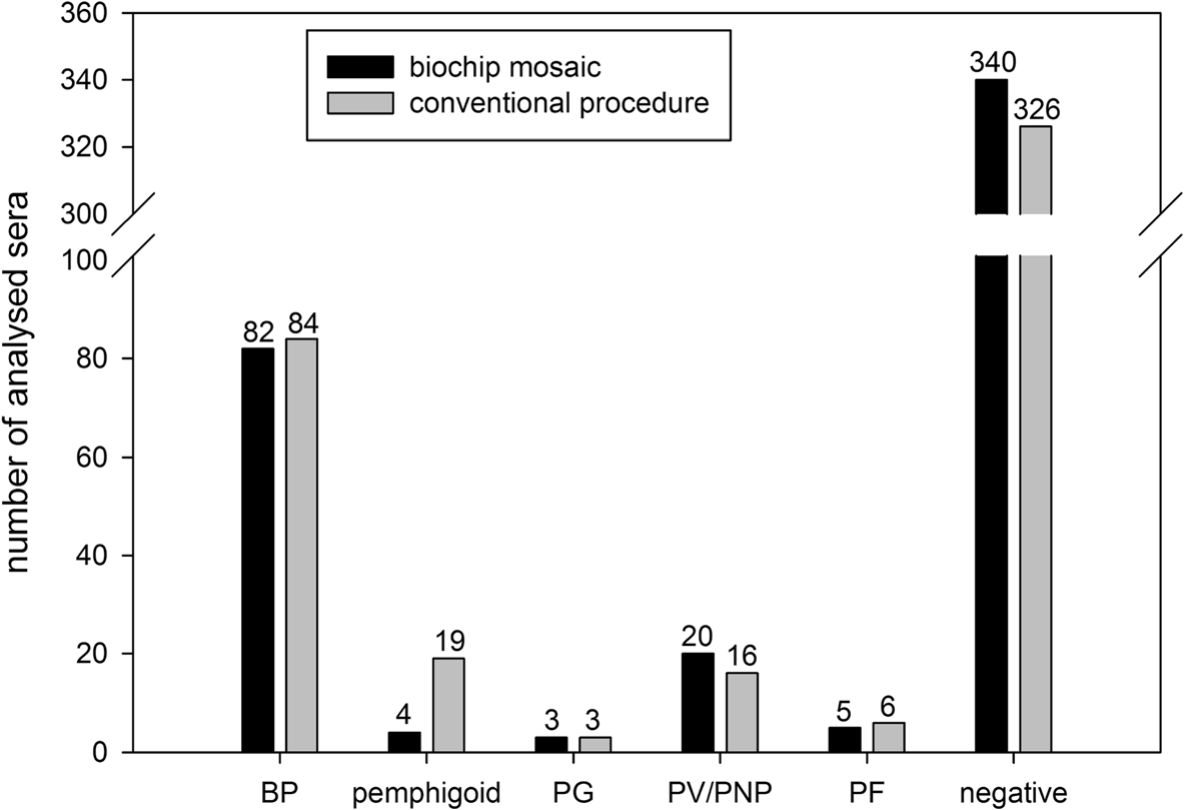
**Evaluation of the BIOCHIP mosaic for the routine diagnosis of autoimmune bullous diseases.** A large panel of consecutive sera (n = 453) from patients with suspected autoimmune bullous disease was subjected to both the BIOCHIP mosaic (black columns) and the routine multi-step procedures of the autoimmune laboratory of the department of dermatology, Luebeck (grey columns; detailed in [38]). Obtained diagnoses were grouped into BP (bullous pemphigoid), “pemphigoid” diseases (including linear IgA disease, p200-pemphigoid, mucous membrane pemphigoid and dermatitis herpetiformis), PV/PNP (pemphigus vulgaris/ paraneoplastic pemphigus), PF (pemphigus foliaceus), and PG (pemphigoid gestationis). PNP and “pemphigoid” sera are further specified in Table 4.

When the 454 consecutive sera from patients with suspected autoimmune blistering disease were subjected to both indirect IF with the BIOCHIP mosaic and the conventional multi-step procedure, concordant diagnoses were obtained in 425 (93.6%) of sera (κ value of 0.88) (Figure 3). BP was diagnosed in 17.8% (n = 81), PV in 3.1% (n = 14), and PF in 1.1% (n = 5). In 322 sera (70.9%), no autoantibody reactivity was found. A high concordance for the diagnosis of BP, PV, PF, and negative sera was found reflected by κ values of 0.97, 0.91 and 0.88, respectively (Figure 3). In 3 sera, both approaches resulted in the diagnosis of pemphigoid gestationis.

In a small portion of sera (n = 21; 4.6%), the conventional multi-step procedure identified additional disorders, including mucous membrane pemphigoid (MMP) (n = 6), linear IgA disease (n = 6), anti-p200 pemphigoid (n = 5), dermatitis herpetiformis (n = 2), and paraneoplastic pemphigus (n = 2). Four of these 21 sera, 2 with MMP, 1 with anti-p200 pemphigoid, and 1 with linear IgA disease, were diagnosed as “pemphigoid” by the BIOCHIP technique based on detection of autoantibodies against BP180 and positive staining on esophagus and/or salt-split skin (Figure 3). The exact results obtained with these sera are detailed in Table 4. After these primary analyses, incongruent results between the conventional multi-step procedure and the BIOCHIP mosaic, which were not explained by the different composition of the two approaches, were seen in 8 (1.8%) sera. For these 8 sera, tests were repeated and appropriate additional analyses were performed, including MBL ELISAs. This post-hoc analysis resolved 5 discrepant results leaving incongruent analyses in 3 (0.7%) sera (Table 4).

**Table 4:** Incongruent results obtained by the BIOCHIP mosaic and the conventional procedure

**Conventional procedure**		**Biochip mosaic**	
**Diagnose**	**Findings**	**Diagnose**	**Findings**
LAD, n = 6	2 with linear IgA deposits on salt-split skin, 4 with anti-LAD-1 IgA reactivity	NS, n = 5; pemphigoid, n = 1	1 with linear IgG/IgA deposits on salt-split skin; 5 without IR
p200, n = 5	linear IgG deposits on salt-split skin and anti-p200 protein/ laminin γ1 reactivity	NS, n = 3; pemphigoid, n = 2	2 with linear IgG/IgA deposits on monkey esophagus and salt split skin; 3 without IR
MMP, n = 6	3 with anti-LAD-1 IgG reactivity, 3 with anti-laminin 332 reactivity	NS, n = 4; pemphigoid, n = 2	2 with linear IgG/IgA deposits on monkey esophagus and salt-split skin; 4 without IR
DH, n = 2	IgA reactivity with endomysium on monkey esophagus and anti-transglutaminase IgA reactivity	NS, n = 2	no IR
PNP, n = 2	Both with anti- Dsg3 reactivity and anti envoplakin reactivity	PV, n = 2	intercellular IgG/IgA deposits on monkey esophagus and Dsg 3-transfected cells
BP, n = 2	positive BP180NC16A ELISA	NS, n = 2	no IR
NS, n = 1	No IR	PV, n = 1	intercellular IgG/IgA deposits on monkey esophagus and Dsg 3-transfected cells

In 21 sera (white background), final diagnosis was based on findings by conventional procedures as explained. In 3 sera (grey background), no final diagnosis could be made. In these cases, direct immunofluorescence microscopy would be required.

IR, immunoreactivity; LAD, linear IgA dermatosis; LAD-1, linear IgA dermatosis antigen 1(soluble ectodomain of BP180); p200, p200-pemphigoid; MMP, mucous membrane pemphigoid; DH, dermatitis herpetiformis; BP, bullous pemphigoid; PV, pemphigus vulgaris; NS, normal serum; PNP, paraneoplastic pemphigus; Dsg 3, desmoglein 3.

## Discussion

The identification of target antigens in the great majority of autoimmune bullous diseases has led to the development of standardized specific and sensitive diagnostic assays, some of them have been commercialized [17,19-23,30,32,34-37,41-45] (Table 1). By the use of these assays, exact diagnosis can be made, which is of both prognostic and therapeutic relevance. Disease’s prognosis may be greatly unfavorable in patients with paraneoplastic pemphigus [46] and anti-laminin-332 mucous membrane pemphigoid [47]. In addition, treatment of epidermolysis bullosa acquisita and pemphigus vulgaris is frequently challenging, while in linear IgA disease, anti-p200 pemphigoid, and in many patients with BP, complete remission can usually be achieved with relatively mild immunosuppression [48-50]. Target-specific diagnostic assays are usually applied in a multi-step approach, starting with a screening test for anti-desmosomal and anti-basal membrane zone antibodies using indirect IF microscopy on monkey esophagus and salt-split skin [15,24-26,38].

In the present study, a BIOCHIP mosaic was composed allowing the simultaneous detection of autoantibodies against the most frequent target antigens to facilitate the diagnosis of bullous skin diseases. Six different substrates were included: monkey esophagus, primate salt-split skin, recombinant BP180 NC16A-4X (antigen dots) and membrane-bound Dsg1 ectodomain, Dsg3 ectodomain, and the C-terminal globular domain of BP230. Additional substrates such as transitional epithelium and cells expressing the immunodominant portions of laminin 332, α6β4 integrin, and type VII collagen that would possibly have improved the detection of autoantibodies in paraneoplastic pemphigus (PNP), MMP, and epidermolysis bullosa acquisita, respectively, were not included due to the rarity of these disorders.

Validation of the BIOCHIP mosaic with sera from BP, PV, and PF patients revealed high sensitivities and specificities comparable with those obtained by conventional approaches e.g. Dsg3 Elisa (97.5-100% and 95.8-97.9%), Dsg1 Elisa (96.0-97.9% and 95.8-97.9%) BP180NC16A Elisa (84.4-89.9% and 97.8-98.9%), and BP230 Elisa (63.0-72.4% and 93–99.5%) [17,19,20,22,30,51,52] (Tables 2 and 3). Previously, indirect IF microscopy of Sf21 insect cells expressing full-length BP180 has shown a sensitivity and specificity of 89% and 100%, respectively, when probed with BP and control sera [53], however, this test is not widely available. The use of HEK293 cells results in the exclusive expression of the mature Dsg1 and Dsg3 forms, which have previously been shown to harbor major pathogenic epitopes on these two ectodomains [54,55]. Cells transfected with the corresponding “empty” plasmid serve as negative control substrates.

To explore the practical application of the BIOCHIP mosaic in the setting of a diagnostic routine laboratory, 454 consecutive sera from patients with suspected autoimmune blistering disease were probed independently using the novel BIOCHIP mosaic IF assay and the routine multi-step diagnostic approach. Results were compared only after the two sets of experiments were completed. High concordance with κ values between 0.88 and 0.97 was observed for the diagnoses of BP, PV, PF, and “negative”.

As expected, the BIOCHIP mosaic did not exactly identify all entities. In fact, in only a small portion of sera (3.7%), the BIOCHIP mosaic was unreactive, while the conventional multistep procedure revealed an autoimmune bullous disease, and in 0.9% of sera, pemphigoid diseases other than BP were detected. The reasons for this comprise the use of an anti-IgA/IgG secondary antibody and the lack of additional target antigens in the BIOCHIP mosaic. The anti-IgA/IgG secondary antibody allows the simultaneous detection of IgG and IgA antibodies which is practical and increases the sensitivity as most sera from patients with BP and MMP [33,56,57]) and many sera from patients with EBA contain IgA autoantibodies in addition to IgG reactivity [58]. However, this secondary antibody does not allow the diagnosis of linear IgA disease and dermatitis herpetiformis. For the diagnosis of anti-laminin 332 mucous membrane pemphigoid, anti-p200/ laminin γ1 pemphigoid, epidermolysis bullosa acquisita, and paraneoplastic pemphigus additional target antigens need to be included in the BIOCHIP mosaic. The complementation of the BIOCHIP mosaic, which may be extended to up to 16 BIOCHIPs in one incubation field, with additional target antigens will, however, only be helpful in a small portion of sera (<5%). In fact, meanwhile, an IF microscopy-based assay employing the membrane-bound immunodominant NC1 domain of type VII collagen expressed in HEK293 cells has been shown as a highly sensitive and specific tool for the serological diagnosis of epidermolysis bullosa acquisita [36]. For the routine application of the current BIOCHIP mosaic we recommend the diagnostic pathway detailed in Additional file 1: Figure S1.

After the initial analysis, discordant results, which were not explained by the different composition of the BIOCHIP mosaic and the multi-step procedure, were seen in only 8 (1.8%) sera. Following post hoc analysis, inconclusive results remained in 3 (0.7%) sera. Since sera had been anonymized, direct IF microscopy could not be performed to clarify the discrepant results in these 3 sera.

It has previously been shown that the investigation of anti-BP230 reactivity in addition to BP180-specific antibodies is only of little diagnostic relevance [59-61]. Since all sera in our relatively small cohort of 42 BP patients contained anti-BP180 antibodies, the diagnostic value of the BP230gC BIOCHIP containing the entire globular C-terminal domain of BP230 could not be assessed. In BP, PV, and PF, autoantibody serum levels were shown to correlate with disease activity [18,62]. Monitoring of serum autoantibody levels during the course of disease may therefore be helpful in the treatment of patients. For the monitoring of autoantibody levels, the available ELISA kits appear to be more convenient than the novel BIOCHIP mosaic.

## Conclusions

BIOCHIP mosaic-based indirect IF technique is useful in screening autoantibodies for routine diagnosis of BP, PV, and PF. Results are concordant compared with the routine multi-step diagnostic procedure. This simple, standardized, and readily available novel tool will further facilitate the diagnosis of autoimmune bullous diseases.

## Abbreviations

BP: Bullous pemphigoid; IF: Immunofluorescence; PF: Pemphigus foliaceus; PV: Pemphigus vulgaris; Dsg: Desmoglein; ELISA: Enzyme linked immunosorbant assay.

## Competing interests

E.S. and D.Z. have a scientific collaboration with EUROIMMUN. K.R., C.P., L.K., K.F., and I-M.B are employed at EUROIMMUN. W.St. is president of EUROIMMUN and principal shareholder. N.vB. and M.K. declared no conflict of interest.

## Authors’ contributions

N.vB. and E.S. analyzed the data and wrote the manuscript. K.R. and IM.B. performed and supervised the BIOCHIP analyses. C.P., L.K., K.F., and W.St. developed the BIOCHIP mosaic technology and drafted the study. M.K. partially performed and supervised the routine multi-step analyses. W.St. and D.Z. advised on the experimental design of the study and revised the manuscript. All authors read and approved the final manuscript.

## Supplementary Material

Additional file 1 ** Figure S1.**Proposed diagnostic pathway following analysis by the BIOCHIP mosaic. LAD-1, linear IgA dermatosis antigen 1(soluble ectodomain of BP180). Click here for file
