# Supplementary figures and images for: Serological diagnosis of autoimmune bullous skin diseases: Prospective comparison of the BIOCHIP mosaic-based indirect immunofluorescence technique with the conventional multi-step single test strategy
Source: Orphanet J Rare Dis. 2012 Aug 9;7:49. doi: 10.1186/1750-1172-7-49 (PMC3533694; doi:10.1186/1750-1172-7-49)

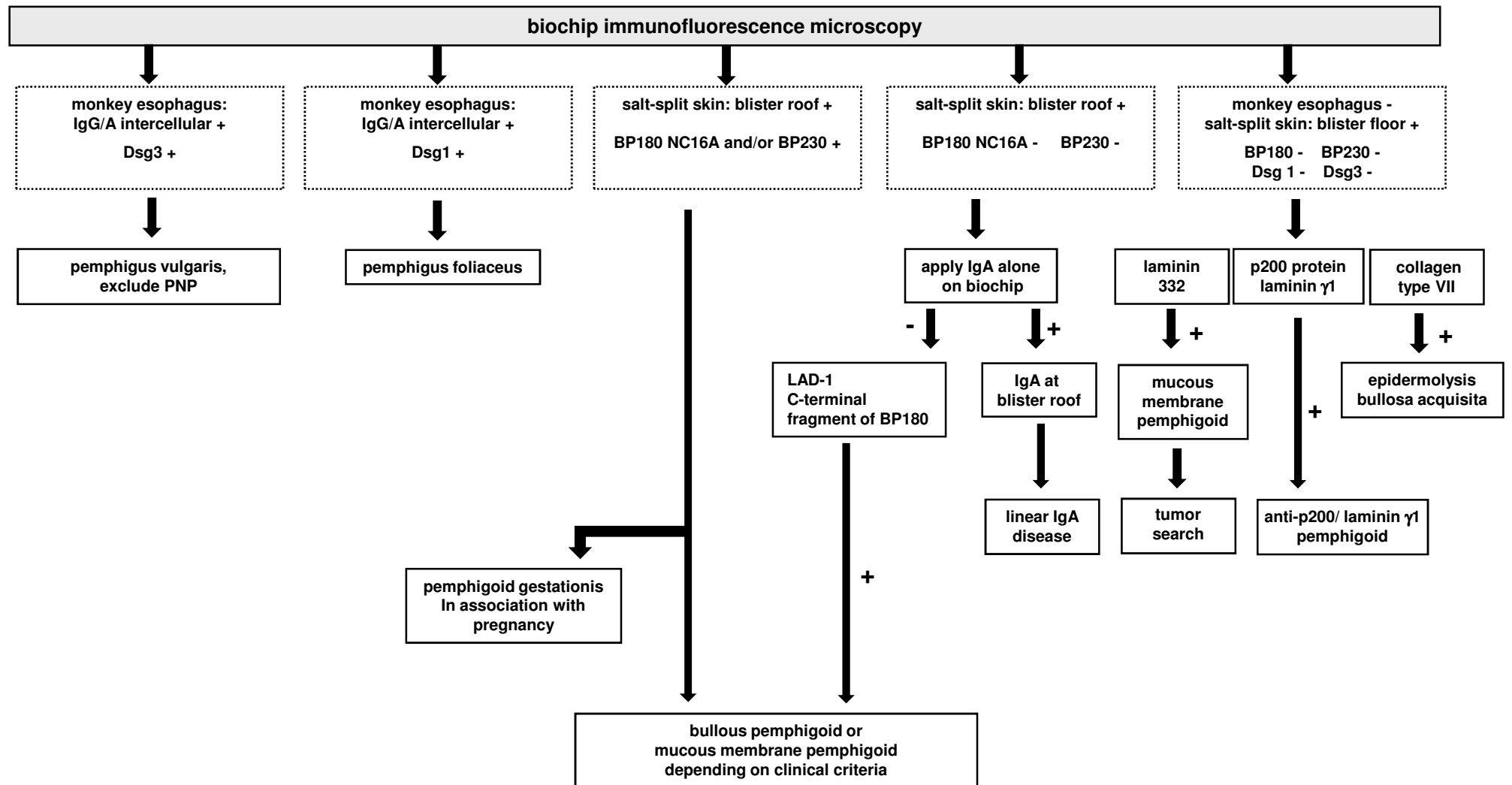

Supplement: Additional file 1 — Figure S1.Proposed diagnostic pathway following analysis by the BIOCHIP mosaic. LAD-1, linear IgA dermatosis antigen 1(soluble ectodomain of BP180). [file 1750-1172-7-49-S1.pdf]
